# Adolescent health in the Sustainable Development Goal era: are we aligned for multisectoral action?

**DOI:** 10.1136/bmjgh-2020-004448

**Published:** 2021-03-16

**Authors:** Asha George, Tanya Jacobs, Rajani Ved, Troy Jacobs, Kumanan Rasanathan, Shehla Abbas Zaidi

**Affiliations:** 1 School of Public Health, University of the Western Cape Faculty of Community and Health Sciences, Cape Town, Western Province, South Africa; 2 National Health Systems Resource Centre, New Delhi, Delhi, India; 3 Global Health Support Initiative III, Rockville, MD, USA; 4 The George Washington University School of Medicine and Health Sciences, Washington, District of Columbia, USA; 5 Health Systems Global member, Phnom Penh, Cambodia; 6 Community Health Sciences, Aga Khan University Faculty of Health Sciences, Karachi, Pakistan

**Keywords:** child health, health policy, health systems

## Abstract

Adolescents are an increasing proportion of low and middle-income country populations. Their coming of age is foundational for health behaviour, as well as social and productive citizenship. We mapped intervention areas for adolescent sexual and reproductive health, including HIV, mental health and violence prevention to sectors responsible for them using a framework that highlights settings, roles and alignment. Out of 11 intervention areas, health is the lead actor for one, and a possible lead actor for two other interventions depending on the implementation context. All other interventions take place outside of the health sector, with the health sector playing a range of bilateral, trilateral supporting roles or in several cases a minimal role. Alignment across the sectors varies from indivisible, enabling or reinforcing to the other extreme of constraining and counterproductive. Governance approaches are critical for brokering these varied relationships and interactions in multisectoral action for adolescent health, to understand the context of such change and to spark, sustain and steer it.

**Summary box**

- The social determinants of health are key in addressing the multiple health burdens faced by adolescents, demanding a far more effective multisectoral response for health than seen to date.
- Out of 11 intervention areas mapped for adolescent health, health is the lead actor for one, and a possible lead actor for two other intervention areas depending on the implementation context.
- All other intervention areas for adolescent health take place outside of the health sector.
- Given the lack of alignment, governance approaches are critical for brokering these varied relationships and interactions in multisectoral action for adolescent health.
- This involves understanding the political context of such multisectoral action, in order to spark, sustain and steer the changes required.

## Introduction

There are just 10 years left to achieve the Sustainable Development Goals (SDGs), with political calls from the highest levels of the UN systems and member states calling for urgent action.^1^ While time is running out for everyone, progress is lagging behind for some groups more than others. For health, accelerating the rate of progress requires scaling up and sustaining effective health interventions, concurrently with improvements in underlying social, economic and environmental conditions that is, the social and structural determinants of health.^2^ The focus of such acceleration, as highlighted once again by the COVID-19 pandemic, must be on those most left behind. This includes adolescents.^1^

Despite being an increasing proportion of low and middle income country populations, adolescents remain a neglected group.^3^ Yet their coming of age is an important foundation for health behaviour, as well as social and productive citizenship for decades to come, shaping our sustainable future. With increasing adolescent population size, the societal contribution adolescents may make is also an important ‘demographic dividend’ for countries.

Adolescents are diverse beings, living in dynamic contexts and facing unprecedented challenges, including a complex burden of disease encompassing diseases of multidimensional poverty and inequality, injuries and non-communicable diseases (NCDs).^4^ The social determinants of health are key in addressing these multiple health burdens, demanding a far more effective multisectoral response for health than seen to date.^5 6^ In this sense all the SDGs, underpin the health and well-being of adolescents, with actions that address poverty, gender inequality, education, criminal justice, housing and food security. This requires extensive work within and across sectors to ensure effective change for adolescents.^7^ But do we understand the implications of what that means for Ministries of Health?

As part of the Countdown 2030 Drivers Technical Working Group, we reviewed the evidence base for adolescent health interventions^8–13^ by drawing from leading reports^3 14^ and existing systematic reviews. By intervention, we mean actions that have a direct or indirect effect on health implemented as projects, programmes or policies. Given the breadth of adolescent health, we focused on three tracer health areas (sexual and reproductive health including HIV, mental health and violence prevention) that make up key aspects of the burden of disease for adolescents and that involve varied actors. We map who is responsible for implementing these interventions across seven areas that emerged from our analysis of the systematic reviews as distinct approaches and settings for adolescent health. Our analysis examines the settings, roles and alignments of different sectors to support multisectoral action for adolescent health within these tracer areas in the SDG era (tables 1 and 2). In doing so, our analysis aims to enable a more nuanced understanding of not only the synergies gained, but also the tensions and precariousness of multisectoral collaboration, and the approaches needed to overcome the challenges involved.^15 16^ By taking stock of what is known and mapping the types of responses required, we flag the importance of not just technical responses but particularly those that take a governance approach to facilitate the different types of relationships and interactions involved in brokering multisectoral collaboration for adolescent health.^17^

**Table 1:** Typology for evaluating roles in multisectoral collaboration for health adapted from Rasanathan *et al*^28^

Health role	Interpretation
Lead	Activity is part of the core mandate of health, collaboration with other sectors is essential for health to deliver on its core mandate.
Bilateral/trilateral	Collaboration is required between two or more sectors to coproduce benefits, manage risks and to maximise health benefits.
Supporting	Collaboration involves initiatives that tackle structural determinants driving health inequalities.
Minimal	Other sector’s core mandates have spill over effects for health.

**Table 2:** Typology for evaluating alignment for multisectoral collaboration adapted from Nilsson *et al*^30^

Alignment	Interpretation
Indivisible	Inextricably linked to the achievement of another goal.
Reinforcing	Aids the achievement of another goal.
Enabling	Creates conditions that further another goal.
Neutral	No significant or negative interactions.
Constraining	Limits options on another goal.
Counteracting	Clashes with another goal.
Cancelling	Makes it impossible to reach another goal.

## Adolescent health: are we aligned for transformative action?

Out of the seven intervention approaches or settings mapped out by our reading of evidence reviews, health is the lead actor for one: the provision of adolescent friendly health services which occurs within health facilities (table 3). It is also a possible lead actor for individual and family based interventions depending on the implementation context. For example, clinical services ranging from therapeutic counselling and voluntary medical male circumcision are a core mandate of the health sector, where health has a lead role.

**Table 3:** Mapping multisectoral implications of adolescent health

#	Intervention approaches/settings	Health issues	Health sector role and actors	Alignment
1	Adolescent-friendly health services at health facilities^11 19 31^	Sexual and Reproductive Health and Rights (SRHR) Mental health	Health sector as lead actor as service provision in health facilities is its core mandate.	Referral linkages between health, education, social services, justice system and peer led networks reinforce the impact of health or constraining if there are not clear systems for referral between the sectors. Good adolescent health services are an enabling factor supporting non-health goals such as school retention.
2	Individual-based/family-based interventions^8 11 19 32^ Therapeutic counselling Voluntary medical male circumcision (VMMC) Parenting programmes Peer mentoring Digital individual and group behavioural interventions Vocational training.	SRHR Mental Health Violence prevention	Health sector as a lead, bilateral/trilateral or minimal actor depending on the intervention or context Clinical services are a core mandate for health. Mental health individual/family based interventions are **co**produced by health, social development, education, sports. VMMC interventions are coproduced by health, education, local government and community/traditional leadership. Violence prevention interventions that are the core business of social development, education, police, has positive spillover effects for health, which is a minimal actor.	Collaboration is reinforcing or enabling, but could also be constraining if there are not clear referral systems, understanding of roles and responsibilities, counteracting if lack of shared vision between various sectors or if other sectors are not effective in preventing violence or when harmful social norms and practices continue or increase.
3	School based interventions^8 9 11 19 32 33^ Provision of health services Health promotion including comprehensive sexuality education Mental health group based interventions Prevention of violence, bullying	SRHR Mental health Violence prevention	Health and education sector as bilateral/trilateral actors for school based interventions School-based interventions are coproduced by health and education	Collaboration between health and education and other sectors could be categorised as reinforcing or enabling but could also be constraining when education and health is not aligned, or counteracting if broader societal contexts are harmful, particularly with regards to gender.
4	Youth centres^11^ Delivering HIV testing, condoms as well as other health services Recreation Vocational training	SRHR	Health sector as a bilateral/trilateral partner or minimal actor depending on the intervention and context Youth centres are coproduced by health and other sectors when delivering health services. Health is a minimal actor when other sectors core business has positive spillover effects for health.	Collaboration between actors could be described as enabling or reinforcing as provision of services through youth centres could contribute to goals of various actors, however it could also be constraining if policies and programmes not aligned.
5	Community mobilisation^8 12 13 19 32^ Prevention of and response to harmful practices, such as female genital mutilation (FGM) and child marriage Promotion of mental health through arts, parent training, child social skills) Prevention of violence against adolescent girls Changing social norms around early marriage and pregnancy	SRHR Mental health Violence prevention	Health sector as a bilateral/trilateral sector, supporting or minimal actor depending on the intervention and context FGM and Cutting (FGM/C) interventions are coproduced by health sector, community and traditional/cultural leadership to change social norms Community based mental health interventions are coproduced by social development, education, and health sectors Changing gender norms to prevent violence against adolescent girls requires support from the health sector for other sectors: social development, criminal justice, gender and/or youth development Gang and street violence prevention, after school dance, leisure or education activities are the core business of other sectors with spillover effects for health which is a minimal actor.	Collaboration could be categorised as multisectoral as preventing harmful practices are inextricably linked to adolescent health constraining if there are not clear systems for referral between the sectors or counteracting if a lack of a shared vision or if conflicts with local norms and practices or when legal and policy environment not supportive.
6	Conditional cash transfers^11^ (CCTs) to adolescents To stay in school To remain Sexually Transmitted Infections (STI) free Unconditional cash transfers (UCTs) to adolescents	SRHR	Health sector as bilateral/trilateral or minimal actor depending on the intervention and context CCTs are coproduced with health if involving delivery of health services UCTs are the core business of other sectors with spillover effects for health, which is a minimal actor	Collaboration could be categorised as reinforcing as interventions which include CCTs have a positive contribution to achieving adolescent health goals and outcomes, and enabling for UCTs as better well-being is supported by health services indirectly.
7	Improve/retain secondary school enrolment and quality of education^20 32^	SRHR Mental health	Health sector as a minimal actor Interventions to improve/retain secondary school enrolment are the core mandate of other sectors with spillover effects for health, which is a minimal actor.	Collaboration could be categorised as enabling as improving retention in secondary school is inextricably linked to adolescent health goals and outcomes. Counteracting when not supported by legal and policy framework and cross cutting gender inequality systems.

However, even the success of voluntary male adolescent medical circumcision rests not just on the clinical service provided, but also in how it is coordinated with the education sector, local government and communities often led by traditional leaders. In this sense the enabling context is coproduced with the health sector. Similarly, mental health interventions for adolescents at individual and family level are coproduced by health with social development, education and sports sectors.

In contrast, violence prevention measures that are aimed at individual adolescents, their families or social groups, are largely the core business of other sectors such as social development, police and education. While vocational training and community policing initiatives aimed at reducing community violence have positive spillover effects for health, health is usually a minimal actor in such endeavours. Similarly, control of tobacco and smoking in adolescents may rest with local government and education sectors, with little involvement of those in the health sector.^18^

In four of the seven intervention approaches or settings (school based interventions, youth centres, community mobilisation and conditional cash transfers), health plays a bilateral or trilateral supporting role as they are involved in coproducing the outcomes. In these kinds of partnerships, roles and responsibilities need to be carefully negotiated, being mindful of the relationships, power and processes at play. However, as mentioned earlier, health may also play a minimal role if the activity is the core mandate of another sector, with benefits for the health sector that are largely through a spillover effect. For example, health benefits enormously from efforts to improve secondary school enrolment and quality of education,^19^ but plays a minimal role because these efforts are the core mandate of the education sector.

Not only does the role of the health sector vary significantly from lead, to supporting or minimal, the alignment across sectors is also varied and will be context specific. Across many of the intervention areas and settings, alignment between health and the other sectors is potentially reinforcing or enabling. For example, good adolescent health services support non-health goals such as school retention.^3^ Effective vocational training and social safety nets can reduce the risk of poverty and violence and therefore reduce morbidity and mortality from injuries for adolescents.^20^

However, alignment is not always positive. Even with school based health interventions, if referral and value systems are not aligned the sectors could be constraining one another or acting in counteracting ways. For example, policies that expel female adolescents from schools for being pregnant, run counter to ensuring inclusive and enabling support systems for such pregnant school-going adolescent mothers. While we did not find any systematic reviews that evaluated interventions that address the commercial marketing of unhealthy foods, tobacco or addictive substances to adolescents, this is clearly an area where health would be at odds with commercial interests.

## Moving multisectoral action for adolescent health from rhetoric to reality

There is increasing documentation of what supports multisectoral action in health, most particularly from a governance lens. The latter is particularly important given the varied roles and alignment signalled above. We propose that there are four key insights from the governance literature on multisectoral action for health that are critical for unleashing the potential for multisectoral action for adolescent health. These include understanding contexts for change, triggers to spark change, capacities to sustain change and relationships to steer change.^21^

### Contexts for change

Multisectoral action is an inherently political endeavour, and therefore, it is critical to understand the contexts in which changing the status quo in favour of multisectoral action for adolescent health takes place. It is critical to map out the different sectors involved, their own hierarchies or power configurations and the interests that drive them. These contextual factors may operate both formally and informally.

In South Africa, no less than six different sectors and related actors published at least 15 adolescent health policies between 2003 and 2018.^22^ Within that context the She Conquers campaign, addressing the structural drivers of adolescent girl’s vulnerability to HIV, started with valuable political leverage from the former deputy President and now current President. It also sought to capitalise on aligning existing significant donor funding to HIV in South Africa.^23^ In Pakistan, the adolescent health agenda is mainly tied to reproductive health focusing on teen pregnancies and undernutrition, supported by scholarly work by a community of academics, clinical doctors and Non-governmental organisations (NGOs) working in maternal and child health. However, adolescent health has not been mainstreamed to other healthcare areas such as tobacco control, NCDs, mental health, missing the opportunity to be framed as a cross-cutting agenda.^18^

A key way of overcoming this complex terrain, including the different starting points involved, is to first understand how different sectors perceive multisectoral collaboration for adolescent health and whether a shared/aligned vision and accountability for common goals can be developed. This foundation is critical for building consensus around mutual goals or framing of the problem and corresponding solutions. In Indonesia, anaemia prevention in adolescent girls was addressed through schools by framing it as a problem affecting school performance and overall well-being, rather than a sexual and reproductive health issue. With this framing it sought buy-in from adolescent girls, teachers and parents as key stakeholders. Evidence on the burden of the problem, the effectiveness and safety of the approach convinced teachers and parents that while it required extra effort it was worthwhile to support the initiative.^24^

### Triggers to spark change

Changing business as usual takes tremendous effort. It is critical to take advantage of triggers or windows of opportunity that further heighten the urgency of multisectoral collaboration for adolescent health. With the She Conquers campaign key survey findings and research highlighting the transmission pathways and the effects of HIV on the economy and on jobs helped to mobilise political capital.^23^ In Malaysia, promotion of the human papilloma virus (HPV) in schools was triggered by the Ministry of Health responding to the World Health Assembly on cervical cancer. It convened an expert committee which reviewed experience from other countries and cost-effectiveness studies in Malaysia.^25^

### Capacities to sustain change

Even once mutual goals are arrived at and triggers motivate change, bureaucracies and systems need to have the capability to respond. This includes having designated human resources, management structures and leadership to support that change, with platforms and teams across sectors to implement change resourced with budgets. The ability to monitor multisectoral action for adolescent health and support continuous learning across the sectors involved is also critical.

In Malaysia, the initiative to address anaemia in adolescent girls built on existing platforms and chose a school based approach given the high rates of coverage of health interventions already achieved through this platform. Previously established operation rooms at national, state and district level for other health conditions became a key way to support the HPV immunisation programme.^25^ Similarly, in Indonesia the school health programme dates back to 1976 and was already endorsed by four ministries with established hierarchies, authorisations and scheduled meetings in place, making it a strong platform for rolling out HPV vaccines.^24^ Lessons in governing multisectoral action stress the importance of using existing structures, unless there is a compelling reason not to.^26^

In the USA, the network that galvanised attention and action on obesity in adolescents (Voices for Healthy Kids) found that investing in dedicated staff trained in stakeholder engagement was critical for fostering strategic relationships, strengthening partner commitments, facilitating fluid communication and building capacity for advocacy. A dedicated portal where grant recipients could access technical assistance providers helped facilitate linkages, but also document and track them. Funding for stakeholder organisations to lead workgroups or taskforces also supported broader coalition building and broader networking.^27^

### Relationships to steer change

Given the complexity of the changes that must be brokered to sustain multisectoral action for adolescent health, it is critical for it to be anchored by relationships underlined by trust and respect to understand divergent needs and ways of working and competencies, including addressing conflicts of interest and managing trade-offs,^26^ to put this into action. Individuals who are able to traverse the boundaries of sectors and institutions—‘boundary spanners’—need to build alliances across agencies to problem solve challenges that are unforeseen. For these decisions to be steered in the right direction, they must not only be consultative across sectors, but also include the voice of adolescents as key agents in driving changes that ultimately benefit them.

In the USA, the Voices for Health Kids initiative recruited health equity and social justice leaders were to its strategic advisory committee. Community involvement was critical for goal setting, strategy selection, garnering broader support and once policies were passed, ensuring their implementation. Through this mobilisation community actors successfully sought resources from existing municipal funding streams. Documentation of success stories further sustained engagement. Overtime the forums to share and build mutual interests, supported by dedicated staff, and continuous learning from evaluations and feedback mechanisms, built relationships of trust. One stakeholder reflected that while messy, there is magic in the messy!”.^27^

Similarly in Malaysia’s HPV school initiative, collaborative relationships evolved beyond the original contractual agreements to more a more nimble and effective ecosystem of partnerships between different parts of government, between government and pharma, between government and the media and between government and parents. Trust and credibility were built through evidence based planning and implementation, bolstered with strategic communication across all partners.^25^

In Indonesia, the success of the school based anaemia prevention programme also was due to relationships built between key actors, even when supported by established mechanisms for coordination. The informal networking supported data sharing and reporting, interest in progress and aided local decision making and accountability for procurement and other matters. Critically, adolescent girls were engaged through multiple phases in the initiative and played a key role in sustaining the initiative given the turnover among teachers.^24^

Ensuring youth engagement is not easy given their heterogeneity and the time and resources required to ensure participation from those most marginalised, as was found in South Africa’s She Conquers campaign. Given that the performance of the campaign varied across provinces and districts, experiences and achievements from stronger locations where shared along with materials and lessons learnt at meetings fostering learning across settings.^23^ In Pakistan, there are also sharp disconnects between youth engagement initiatives and adolescent health initiatives. Youth engagement is politically championed by the highest level in Pakistan and supported by an increasing number of programmes many of which are managed by youth, however, adolescent health remains an area operationally confined to research and a few donor supported government programmes failing to draw on to youth engagement and discourse platforms.^18^

## Conclusion

Governance matters and is a priority for the alignment of interventions in the SDG era. Correspondingly, both the technical and political aspects of multisectoral action for adolescent health must be prioritised. Looking across the evidence-based interventions that address adolescent health, it is clear from our mapping that multisectoral approaches are required for the majority of them although the exact nature of these approaches will vary across contexts. As a consequence, we can no longer treat adolescent health as being mainly the concern of the health sector; sectors ranging from education, social development, transport and communications, water and sanitation, and safety play vital roles. The challenges involved are precisely because health is not always the lead sector supporting multisectoral action for adolescent health in the SDG era and the alignment of sectors is not always positive.

Rasanathan *et al*^28^ notes that multisectoral collaboration for health is not equivalent to health imperialism. Conventional modes of command and control hierarchy ingrained in Ministries of Health may be particularly ill suited to facilitate multisectoral action for adolescent health involving both public and private health sectors and collaboration with other sectors. To move from rhetoric to reality, we outline key governance lessons critical for supporting multisectoral action by understanding (1) the political ecosystems or contexts for change, (2) triggers to spark change, (3) capacities to sustain change and (4) relationships to steer change. Further research is needed to enhance the understandings of the political ecosystems, mapping stakeholders and institutional architecture and power relations, identifying champions and alliances and reviewing and adapting the typologies noted above.^29^ Further, reviewing and building capacity at individual, organisational and systems level for distributed leadership and management and strengthening capacity for collaboration, as well as adolescent-responsive implementation, is needed going forward.

Rather than assume that Ministries of Health are natural leads for adolescent health, explicitly examining the governance implications may help sustain multisectoral action. It takes cognizance of the humility required in addressing the complexities involved and foregrounds the voice of adolescents given that their diversity and agency cannot continue to be ignored.
